# Supplementary material for: A Likelihood Approach for Real-Time Calibration of Stochastic Compartmental Epidemic Models
Source: PLoS Comput Biol. 2017 Jan 17;13(1):e1005257. doi: 10.1371/journal.pcbi.1005257 (PMC5240920; doi:10.1371/journal.pcbi.1005257)
Supplement: S1 File — (TAR.GZ) [file pcbi.1005257.s014.tar.gz › HSPH_Online-SI-Revision/IPoi09/n100-extreme/n100-extreme_BENCH2_table-8.pdf]

| | | | | | | | | | | | | |
|---|---|---|---|---|---|---|---|---|---|---|---|---|
| 1 | newly 3 weeks spec | 1767. | 2118.49 | {154., 4836.} | 82.3884 | 0.427844 | newly 3 weeks cumul | 3085. | 3668.52 | {257., 8321.} | 84.3153 | 0.438507 |
| 2 | newly 3 weeks spec | 2838. | 7740.94 | {5048., 9445.} | 175.845 | 0.431296 | newly 3 weeks cumul | 5925. | 21552.7 | {9974., 27436.} | 267.258 | 0.54317 |
| 3 | newly 3 weeks spec | 1557. | 2968.49 | {457., 5572.} | 119.347 | 0.37198 | newly 3 weeks cumul | 2607. | 5033.22 | {726., 9553.} | 122.995 | 0.376936 |
| 4 | newly 3 weeks spec | 302. | 391.767 | {0., 747.} | 118.985 | 0.908376 | newly 3 weeks cumul | 421. | 536.683 | {1., 1065.} | 113.551 | 0.829978 |
| 5 | newly 3 weeks spec | 841. | 2338.83 | {0., 5514.} | 234.252 | 1.22841 | newly 3 weeks cumul | 1316. | 4838. | {0., 12726.} | 322.18 | 1.27923 |
| 6 | newly 3 weeks spec | 1468. | 4445.95 | {959., 8609.} | 217.567 | 0.458091 | newly 3 weeks cumul | 2440. | 8514.29 | {1571., 17253.} | 263.288 | 0.496783 |
| 7 | newly 3 weeks spec | 1624. | 769.761 | {200., 1935.} | 60.2805 | 0.504252 | newly 3 weeks cumul | 2704. | 1437.7 | {342., 3577.} | 59.0391 | 0.470729 |
| 8 | newly 3 weeks spec | 1359. | 3397.2 | {468., 7357.} | 182.247 | 0.547364 | newly 3 weeks cumul | 2461. | 6192.22 | {793., 14199.} | 186.134 | 0.549852 |
| 9 | newly 3 weeks spec | 953. | 514.85 | {98., 980.} | 54.0206 | 0.452924 | newly 3 weeks cumul | 1486. | 923.05 | {169., 1908.} | 53.7607 | 0.417498 |
| 10 | newly 3 weeks spec | 629. | 1780.56 | {183., 3337.} | 210.587 | 0.584109 | newly 3 weeks cumul | 952. | 2452.17 | {257., 4667.} | 186.5 | 0.540947 |
| 11 | newly 3 weeks spec | 2514. | 4858.24 | {1250., 6251.} | 109.256 | 0.355359 | newly 3 weeks cumul | 4845. | 12848.3 | {2221., 19461.} | 181.82 | 0.47273 |
| 12 | newly 3 weeks spec | 1011. | 251.305 | {28., 632.} | 75.3642 | 0.827351 | newly 3 weeks cumul | 1634. | 485.085 | {53., 1222.} | 71.3835 | 0.754688 |
| 13 | newly 3 weeks spec | 2062. | 5047.67 | {1504., 8089.} | 159.566 | 0.423754 | newly 3 weeks cumul | 3828. | 10757.7 | {2849., 18274.} | 195.779 | 0.466893 |
| 14 | newly 3 weeks spec | 207. | 384.818 | {57., 727.} | 119.807 | 0.614412 | newly 3 weeks cumul | 282. | 927.364 | {223., 1766.} | 249.936 | 0.587612 |
| 15 | newly 3 weeks spec | 874. | 747.644 | {60., 2106.} | 75.3772 | 0.585942 | newly 3 weeks cumul | 1434. | 1259.95 | {89., 3400.} | 77.3739 | 0.584099 |
| 16 | newly 3 weeks spec | 582. | 115. | {6., 306.} | 81.1991 | 1.09295 | newly 3 weeks cumul | 805. | 213.737 | {13., 538.} | 75.8636 | 0.950218 |
| 17 | newly 3 weeks spec | 2320. | 2146.6 | {210., 4758.} | 61.4463 | 0.418415 | newly 3 weeks cumul | 4411. | 4088.4 | {359., 9451.} | 64.0772 | 0.434187 |
| 18 | newly 3 weeks spec | 1347. | 965.174 | {153., 2434.} | 58.3261 | 0.441637 | newly 3 weeks cumul | 2379. | 1805.25 | {259., 4442.} | 59.7139 | 0.43388 |
| 19 | newly 3 weeks spec | 927. | 415.97 | {99., 1028.} | 66.2482 | 0.569367 | newly 3 weeks cumul | 1494. | 743.333 | {164., 1809.} | 65.1069 | 0.532374 |
| 20 | newly 3 weeks spec | 53. | 60.8906 | {0., 181.} | 132.223 | 1.06783 | newly 3 weeks cumul | 74. | 92.8125 | {0., 253.} | 133.826 | 0.969951 |
| 21 | newly 3 weeks spec | 845. | 1447.98 | {114., 3209.} | 119.239 | 0.539629 | newly 3 weeks cumul | 1345. | 2411.76 | {189., 5162.} | 126.638 | 0.523085 |
| 22 | newly 3 weeks spec | 804. | 1033.16 | {55., 2233.} | 100.144 | 0.636829 | newly 3 weeks cumul | 1237. | 1740.05 | {104., 3869.} | 111.391 | 0.606256 |
| 23 | newly 3 weeks spec | 2606. | 3697.52 | {979., 7765.} | 75.5009 | 0.291355 | newly 3 weeks cumul | 5006. | 7844.57 | {1779., 17601.} | 90.885 | 0.317531 |
| 24 | newly 3 weeks spec | 1803. | 1557.9 | {238., 3267.} | 56.8127 | 0.363775 | newly 3 weeks cumul | 3172. | 3204.67 | {462., 6927.} | 65.1251 | 0.361875 |
| 25 | newly 3 weeks spec | 784. | 133.691 | {21., 321.} | 82.9476 | 1.00982 | newly 3 weeks cumul | 1188. | 274.364 | {48., 618.} | 77.2727 | 0.875857 |
| 26 | newly 3 weeks spec | 2337. | 4509.47 | {821., 8448.} | 121.287 | 0.370236 | newly 3 weeks cumul | 4561. | 9439.48 | {1488., 18617.} | 137.001 | 0.393974 |
| 27 | newly 3 weeks spec | 3178. | 8153.21 | {4896., 11275.} | 158.936 | 0.39549 | newly 3 weeks cumul | 6240. | 20479.4 | {9233., 27007.} | 231.004 | 0.48835 |
| 28 | newly 3 weeks spec | 1642. | 7650.66 | {1984., 13470.} | 377.183 | 0.649523 | newly 3 weeks cumul | 2833. | 13233.2 | {3031., 25076.} | 379.237 | 0.639819 |
| 29 | newly 3 weeks spec | 2186. | 4274.26 | {1355., 6337.} | 105.034 | 0.297046 | newly 3 weeks cumul | 4260. | 9841.11 | {2496., 16196.} | 140.56 | 0.35024 |
| 30 | newly 3 weeks spec | 404. | 5022.33 | {0., 12273.} | 1178.94 | 1.41465 | newly 3 weeks cumul | 607. | 7202.3 | {0., 17521.} | 1122.34 | 1.41805 |
| 31 | newly 3 weeks spec | 169. | 188.2 | {0., 179.} | 178.142 | 2.11451 | newly 3 weeks cumul | 246. | 228.88 | {0., 237.} | 158.146 | 1.94722 |
| 32 | newly 3 weeks spec | 874. | 2631.98 | {365., 5900.} | 217.75 | 0.43666 | newly 3 weeks cumul | 1385. | 3851.39 | {553., 8506.} | 196.923 | 0.41129 |
| 33 | newly 3 weeks spec | 1347. | 5593.31 | {331., 10100.} | 333.828 | 0.711974 | newly 3 weeks cumul | 2306. | 11656.3 | {473., 24455.} | 424.19 | 0.767445 |
| 34 | newly 3 weeks spec | 2211. | 5303.23 | {888., 9004.} | 160.124 | 0.442191 | newly 3 weeks cumul | 4652. | 11755.3 | {1636., 21690.} | 174.826 | 0.460645 |
| 35 | newly 3 weeks spec | 845. | 1751.95 | {304., 3246.} | 135.6 | 0.421346 | newly 3 weeks cumul | 1377. | 3011.3 | {506., 5695.} | 145.798 | 0.431859 |
| 36 | newly 3 weeks spec | 2415. | 5446.29 | {3775., 7101.} | 127.278 | 0.343044 | newly 3 weeks cumul | 4659. | 16116.6 | {12673., 20007.} | 247.338 | 0.52475 |
| 37 | newly 3 weeks spec | 569. | 713.145 | {55., 1810.} | 85.2857 | 0.530249 | newly 3 weeks cumul | 794. | 1030.75 | {96., 2554.} | 88.0736 | 0.512242 |
| 38 | newly 3 weeks spec | 174. | 539.818 | {0., 1275.} | 290.961 | 1.4748 | newly 3 weeks cumul | 245. | 1063.14 | {0., 2263.} | 408.108 | 1.34201 |
| 39 | newly 3 weeks spec | 1689. | 2162.62 | {489., 4596.} | 74.0347 | 0.30332 | newly 3 weeks cumul | 3181. | 4061.88 | {846., 8599.} | 77.1422 | 0.315867 |
| 40 | newly 3 weeks spec | 2733. | 5097.81 | {2485., 7104.} | 97.8429 | 0.292579 | newly 3 weeks cumul | 5234. | 12135.6 | {4533., 19524.} | 144.981 | 0.366561 |
| 41 | newly 3 weeks spec | 2085. | 6001.35 | {1909., 8731.} | 189.772 | 0.421859 | newly 3 weeks cumul | 3973. | 13173.5 | {3329., 21997.} | 235.39 | 0.46589 |
| 42 | newly 3 weeks spec | 641. | 4763.37 | {597., 9133.} | 656.315 | 0.910171 | newly 3 weeks cumul | 992. | 7271.41 | {816., 15252.} | 647.55 | 0.907005 |
| 43 | newly 3 weeks spec | 1358. | 499.824 | {97., 1116.} | 65.0892 | 0.603835 | newly 3 weeks cumul | 2359. | 977.603 | {180., 2187.} | 63.0682 | 0.562408 |
| 44 | newly 3 weeks spec | 944. | 2890.24 | {361., 6845.} | 231.245 | 0.485452 | newly 3 weeks cumul | 1464. | 4507.13 | {506., 11002.} | 233.554 | 0.484743 |
| 45 | newly 3 weeks spec | 599. | 60.1077 | {6., 147.} | 89.9653 | 1.2697 | newly 3 weeks cumul | 910. | 112.415 | {13., 264.} | 87.6467 | 1.17961 |
| 46 | newly 3 weeks spec | 564. | 176.877 | {28., 486.} | 71.0065 | 0.712946 | newly 3 weeks cumul | 898. | 305.585 | {47., 794.} | 69.2496 | 0.674415 |
| 47 | newly 3 weeks spec | 765. | 3231.78 | {384., 7762.} | 342.993 | 0.704036 | newly 3 weeks cumul | 1158. | 4433.14 | {508., 10829.} | 304.504 | 0.675137 |
| 48 | newly 3 weeks spec | 268. | 435.328 | {51., 1187.} | 123.163 | 0.521914 | newly 3 weeks cumul | 370. | 652.203 | {75., 1790.} | 132.842 | 0.497563 |
| 49 | newly 3 weeks spec | 151. | 3207.14 | {0., 10728.} | 2135.58 | 2.09771 | newly 3 weeks cumul | 195. | 3739.71 | {0., 12511.} | 1926.79 | 2.03321 |
| 50 | newly 3 weeks spec | 2772. | 2282.21 | {550., 4705.} | 52.0245 | 0.322615 | newly 3 weeks cumul | 5085. | 4378.39 | {991., 9143.} | 55.6495 | 0.331057 |

| | | | | | | | | | | | | |
|---|---|---|---|---|---|---|---|---|---|---|---|---|
| 1 | newly 5 weeks spec | 5804. | 6081.58 | {813., 11204.} | 55.0359 | 0.324203 | newly 5 weeks cumul | 12241. | 13592.5 | {1420., 28020.} | 66.7092 | 0.367546 |
| 2 | newly 5 weeks spec | 5914. | 6765.4 | {4276., 9307.} | 28.1504 | 0.1115 | newly 5 weeks cumul | 16050. | 35810.4 | {26122., 42223.} | 125.543 | 0.347446 |
| 3 | newly 5 weeks spec | 6571. | 8420.36 | {2377., 12618.} | 53.9124 | 0.238986 | newly 5 weeks cumul | 12454. | 18914.1 | {4173., 31756.} | 78.6605 | 0.29647 |
| 4 | newly 5 weeks spec | 3492. | 3192.9 | {0., 8006.} | 93.4737 | 1.15537 | newly 5 weeks cumul | 5026. | 4967.9 | {1., 11431.} | 98.4103 | 1.05591 |
| 5 | newly 5 weeks spec | 3644. | 3468.56 | {0., 6462.} | 56.4642 | 1.25474 | newly 5 weeks cumul | 6776. | 11334.6 | {0., 21841.} | 125.766 | 1.27372 |
| 6 | newly 5 weeks spec | 5118. | 8573.79 | {3711., 11790.} | 79.782 | 0.260375 | newly 5 weeks cumul | 10351. | 23694.6 | {7180., 39552.} | 141.869 | 0.365929 |
| 7 | newly 5 weeks spec | 5875. | 2208.78 | {615., 4752.} | 62.9358 | 0.545823 | newly 5 weeks cumul | 11979. | 4976.57 | {1290., 11398.} | 60.2939 | 0.521195 |
| 8 | newly 5 weeks spec | 5170. | 7725.72 | {2023., 12212.} | 79.8966 | 0.396337 | newly 5 weeks cumul | 10407. | 19427.2 | {3775., 36603.} | 118.473 | 0.471181 |
| 9 | newly 5 weeks spec | 4055. | 1757.55 | {384., 3375.} | 57.4809 | 0.499532 | newly 5 weeks cumul | 7477. | 3644.98 | {782., 7546.} | 54.4138 | 0.465173 |
| 10 | newly 5 weeks spec | 4393. | 10929. | {2100., 17869.} | 170.253 | 0.567279 | newly 5 weeks cumul | 6990. | 18530.9 | {3023., 31674.} | 187.867 | 0.570885 |
| 11 | newly 5 weeks spec | 6044. | 4426.65 | {2160., 6342.} | 29.3427 | 0.18524 | newly 5 weeks cumul | 15023. | 22249.9 | {8376., 26607.} | 62.9188 | 0.243097 |
| 12 | newly 5 weeks spec | 4508. | 710.763 | {102., 1728.} | 84.2333 | 0.997977 | newly 5 weeks cumul | 8400. | 1621.95 | {200., 4080.} | 80.6911 | 0.919417 |
| 13 | newly 5 weeks spec | 4926. | 7793.52 | {4120., 9994.} | 70.3452 | 0.263185 | newly 5 weeks cumul | 12079. | 25144.1 | {9346., 37759.} | 121.623 | 0.364701 |
| 14 | newly 5 weeks spec | 1855. | 540.636 | {4., 1054.} | 70.8552 | 1.05127 | newly 5 weeks cumul | 2833. | 1946.36 | {259., 3615.} | 47.0398 | 0.465823 |
| 15 | newly 5 weeks spec | 4922. | 2873.14 | {332., 6429.} | 58.374 | 0.585058 | newly 5 weeks cumul | 8480. | 5657.34 | {560., 14346.} | 63.1802 | 0.592429 |
| 16 | newly 5 weeks spec | 4823. | 376.474 | {10., 1023.} | 92.1942 | 1.53874 | newly 5 weeks cumul | 7337. | 801.965 | {35., 2066.} | 89.0696 | 1.37421 |
| 17 | newly 5 weeks spec | 5419. | 4968.18 | {745., 9140.} | 46.4795 | 0.320505 | newly 5 weeks cumul | 13371. | 12478.1 | {1504., 25228.} | 54.5426 | 0.366672 |
| 18 | newly 5 weeks spec | 4153. | 2667.32 | {489., 6186.} | 50.7229 | 0.415908 | newly 5 weeks cumul | 9012. | 6115.87 | {1027., 14711.} | 54.3171 | 0.425602 |
| 19 | newly 5 weeks spec | 4254. | 1367.92 | {336., 3293.} | 69.6297 | 0.656876 | newly 5 weeks cumul | 7918. | 2873.05 | {650., 6942.} | 68.0974 | 0.623862 |
| 20 | newly 5 weeks spec | 1059. | 475.984 | {0., 1745.} | 99.3257 | 1.80565 | newly 5 weeks cumul | 1395. | 738.266 | {0., 2576.} | 100.698 | 1.56075 |
| 21 | newly 5 weeks spec | 4282. | 5142.08 | {663., 9253.} | 67.5999 | 0.470684 | newly 5 weeks cumul | 7677. | 10456.2 | {1141., 22419.} | 84.0197 | 0.485716 |
| 22 | newly 5 weeks spec | 4322. | 3909.25 | {290., 9062.} | 61.1359 | 0.577217 | newly 5 weeks cumul | 7480. | 7785.55 | {543., 17700.} | 73.6169 | 0.573729 |
| 23 | newly 5 weeks spec | 5905. | 6320.36 | {2890., 9143.} | 35.0245 | 0.177184 | newly 5 weeks cumul | 15055. | 19180.7 | {6421., 35799.} | 58.6048 | 0.24755 |
| 24 | newly 5 weeks spec | 5220. | 3249.17 | {672., 6032.} | 45.0728 | 0.354121 | newly 5 weeks cumul | 11528. | 8782.5 | {1557., 17663.} | 50.0815 | 0.351519 |
| 25 | newly 5 weeks spec | 4655. | 350.418 | {56., 853.} | 92.4722 | 1.34368 | newly 5 weeks cumul | 7743. | 840.8 | {146., 2032.} | 89.1412 | 1.18603 |
| 26 | newly 5 weeks spec | 5505. | 7321.92 | {2752., 9962.} | 55.346 | 0.226197 | newly 5 weeks cumul | 13758. | 22831.3 | {5887., 37792.} | 91.5453 | 0.312377 |
| 27 | newly 5 weeks spec | 6900. | 7768.97 | {3429., 10604.} | 30.1718 | 0.129799 | newly 5 weeks cumul | 18218. | 36696.4 | {25551., 48779.} | 103.026 | 0.296762 |
| 28 | newly 5 weeks spec | 6075. | 13586.2 | {7361., 16702.} | 131.444 | 0.375849 | newly 5 weeks cumul | 12201. | 38401.2 | {18044., 55668.} | 223.446 | 0.509765 |
| 29 | newly 5 weeks spec | 5160. | 5745.81 | {3234., 6739.} | 22.4325 | 0.094147 | newly 5 weeks cumul | 12858. | 20764.9 | {7918., 28898.} | 70.7754 | 0.227304 |
| 30 | newly 5 weeks spec | 3670. | 13074.9 | {0., 23031.} | 291.079 | 1.22063 | newly 5 weeks cumul | 5636. | 30427.1 | {0., 56363.} | 474.824 | 1.37872 |
| 31 | newly 5 weeks spec | 2181. | 1879.18 | {0., 2344.} | 156.291 | 2.90049 | newly 5 weeks cumul | 3075. | 2932.35 | {0., 3251.} | 164.966 | 2.63974 |
| 32 | newly 5 weeks spec | 4137. | 11544.1 | {3850., 17210.} | 184.184 | 0.423011 | newly 5 weeks cumul | 7503. | 21712.5 | {5705., 38353.} | 197.796 | 0.431631 |
| 33 | newly 5 weeks spec | 4307. | 7269.72 | {1640., 10386.} | 86.1686 | 0.411698 | newly 5 weeks cumul | 9125. | 25829.8 | {2812., 41144.} | 200.795 | 0.604888 |
| 34 | newly 5 weeks spec | 4361. | 7464.52 | {2567., 9407.} | 86.2023 | 0.306155 | newly 5 weeks cumul | 12123. | 25816.8 | {5821., 39609.} | 131.068 | 0.394008 |
| 35 | newly 5 weeks spec | 3852. | 5496.68 | {1438., 9564.} | 72.6103 | 0.336278 | newly 5 weeks cumul | 7034. | 11800.7 | {2637., 21365.} | 96.3939 | 0.380028 |
| 36 | newly 5 weeks spec | 5307. | 3818.48 | {1289., 6701.} | 38.3671 | 0.247059 | newly 5 weeks cumul | 13623. | 24715.1 | {19320., 30116.} | 82.1755 | 0.253464 |
| 37 | newly 5 weeks spec | 3995. | 4968.42 | {427., 11144.} | 78.4009 | 0.545363 | newly 5 weeks cumul | 6432. | 8047.33 | {668., 18795.} | 80.6826 | 0.544052 |
| 38 | newly 5 weeks spec | 2064. | 1176.36 | {0., 3592.} | 76.6517 | 1.85303 | newly 5 weeks cumul | 2893. | 3069. | {0., 9402.} | 105.609 | 1.51494 |
| 39 | newly 5 weeks spec | 4543. | 5256.6 | {1795., 9160.} | 48.3713 | 0.219514 | newly 5 weeks cumul | 10645. | 12823.9 | {3547., 24738.} | 60.2992 | 0.26038 |
| 40 | newly 5 weeks spec | 6145. | 5446.29 | {3775., 7101.} | 18.7524 | 0.100724 | newly 5 weeks cumul | 15669. | 23154.4 | {14743., 29862.} | 57.9753 | 0.204107 |
| 41 | newly 5 weeks spec | 5334. | 6947.95 | {4533., 8687.} | 33.6174 | 0.121692 | newly 5 weeks cumul | 12888. | 27133.7 | {13430., 35315.} | 111.291 | 0.305499 |
| 42 | newly 5 weeks spec | 4341. | 12800.1 | {4214., 19823.} | 206.09 | 0.6292 | newly 5 weeks cumul | 7191. | 29317.7 | {6258., 49272.} | 319.667 | 0.759261 |
| 43 | newly 5 weeks spec | 4809. | 1327.66 | {225., 2913.} | 72.3921 | 0.701619 | newly 5 weeks cumul | 9757. | 3135.28 | {546., 6930.} | 68.1802 | 0.645349 |
| 44 | newly 5 weeks spec | 6185. | 9930.75 | {2318., 15740.} | 84.0292 | 0.302443 | newly 5 weeks cumul | 10162. | 20450.5 | {3681., 39577.} | 125.175 | 0.370464 |
| 45 | newly 5 weeks spec | 4808. | 202.538 | {14., 521.} | 95.7875 | 1.70711 | newly 5 weeks cumul | 7429. | 425.092 | {32., 938.} | 94.2779 | 1.54726 |
| 46 | newly 5 weeks spec | 3017. | 725.2 | {103., 2015.} | 75.9629 | 0.821202 | newly 5 weeks cumul | 5294. | 1393.6 | {194., 3938.} | 74.1838 | 0.777902 |
| 47 | newly 5 weeks spec | 3839. | 14916. | {4085., 21149.} | 302.003 | 0.731479 | newly 5 weeks cumul | 6738. | 27820.8 | {5915., 47603.} | 327.553 | 0.75228 |
| 48 | newly 5 weeks spec | 2437. | 2704.48 | {419., 6845.} | 82.7599 | 0.542552 | newly 5 weeks cumul | 3631. | 4489.73 | {652., 11605.} | 91.863 | 0.527577 |
| 49 | newly 5 weeks spec | 3032. | 8071.13 | {0., 27675.} | 282.881 | 2.59515 | newly 5 weeks cumul | 3868. | 18679.9 | {0., 66809.} | 498.841 | 2.61012 |
| 50 | newly 5 weeks spec | 6948. | 5218.78 | {1821., 9231.} | 36.8301 | 0.245508 | newly 5 weeks cumul | 16731. | 13193.7 | {3842., 25348.} | 44.6465 | 0.285265 |

| | | | | | | |
|---|---|---|---|---|---|---|
| 1 | newly 62 weeks cumul | 91729. | 87684. | {82896., 91970.} | 4.69609 | 0.02158 |
| 2 | newly 58 weeks cumul | 82551. | 58618.2 | {45358., 72687.} | 28.9915 | 0.155572 |
| 3 | newly 63 weeks cumul | 90095. | 91262.1 | {87967., 93622.} | 2.99021 | 0.0130155 |
| 4 | newly 67 weeks cumul | 90300. | 69305.7 | {1., 93479.} | 24.57 | 0.848544 |
| 5 | newly 64 weeks cumul | 88461. | 26385.4 | {0., 43555.} | 70.1729 | 1.48966 |
| 6 | newly 62 weeks cumul | 91404. | 83537.3 | {74207., 92448.} | 9.07402 | 0.0435835 |
| 7 | newly 60 weeks cumul | 90884. | 89605.5 | {84975., 92456.} | 2.98549 | 0.0132793 |
| 8 | newly 62 weeks cumul | 92361. | 85263.8 | {76556., 91993.} | 7.75525 | 0.121845 |
| 9 | newly 62 weeks cumul | 92411. | 85643.4 | {75790., 93276.} | 7.7211 | 0.0372538 |
| 10 | newly 66 weeks cumul | 75317. | 84765. | {79315., 93371.} | 19.8296 | 0.227276 |
| 11 | newly 60 weeks cumul | 84746. | 35867.4 | {27496., 41228.} | 57.6766 | 0.377563 |
| 12 | newly 61 weeks cumul | 92398. | 84877.5 | {80425., 90734.} | 8.13924 | 0.0373371 |
| 13 | newly 59 weeks cumul | 90371. | 77618.8 | {71363., 86360.} | 14.1109 | 0.0678844 |
| 14 | newly 64 weeks cumul | 71218. | 10516.3 | {259., 15271.} | 85.2337 | 1.17249 |
| 15 | newly 64 weeks cumul | 79509. | 78826.2 | {58888., 93894.} | 15.2367 | 0.162241 |
| 16 | newly 64 weeks cumul | 91266. | 81127.6 | {71550., 92615.} | 11.5744 | 0.247058 |
| 17 | newly 59 weeks cumul | 87876. | 89701.6 | {88396., 90821.} | 2.45677 | 0.0105495 |
| 18 | newly 60 weeks cumul | 79943. | 89697.3 | {85886., 92891.} | 12.5425 | 0.0510916 |
| 19 | newly 62 weeks cumul | 93229. | 89521.8 | {80848., 93140.} | 4.02681 | 0.0184726 |
| 20 | newly 67 weeks cumul | 91568. | 65170.3 | {0., 92561.} | 29.2867 | 1.21957 |
| 21 | newly 64 weeks cumul | 83732. | 73046.2 | {54175., 91738.} | 19.5485 | 0.27707 |
| 22 | newly 65 weeks cumul | 84795. | 66735.3 | {42253., 89342.} | 24.5604 | 0.333132 |
| 23 | newly 58 weeks cumul | 84036. | 82227.4 | {70532., 88984.} | 5.62161 | 0.0259413 |
| 24 | newly 58 weeks cumul | 92404. | 82768.9 | {74595., 88295.} | 10.4271 | 0.0486056 |
| 25 | newly 61 weeks cumul | 89551. | 81526. | {73642., 87910.} | 8.96141 | 0.0444147 |
| 26 | newly 59 weeks cumul | 89914. | 78154.3 | {72216., 90514.} | 13.494 | 0.0639675 |
| 27 | newly 60 weeks cumul | 90939. | 58829.9 | {39963., 77408.} | 35.3084 | 0.198644 |
| 28 | newly 64 weeks cumul | 93780. | 83458.2 | {73457., 92320.} | 11.0065 | 0.0549376 |
| 29 | newly 58 weeks cumul | 82934. | 51453.3 | {46057., 67298.} | 37.9587 | 0.210712 |
| 30 | newly 67 weeks cumul | 93251. | 61270.2 | {0., 88483.} | 34.2954 | 1.04281 |
| 31 | newly 68 weeks cumul | 88759. | 40862.6 | {0., 92226.} | 55.5766 | 2.75239 |
| 32 | newly 65 weeks cumul | 77539. | 89292.4 | {84271., 93572.} | 15.8618 | 0.0637959 |
| 33 | newly 63 weeks cumul | 92452. | 51901.7 | {44888., 65846.} | 43.861 | 0.390616 |
| 34 | newly 58 weeks cumul | 74850. | 73444.7 | {67367., 76807.} | 4.92124 | 0.0216797 |
| 35 | newly 63 weeks cumul | 93735. | 85857.1 | {77642., 92641.} | 8.40442 | 0.130713 |
| 36 | newly 61 weeks cumul | 83417. | 32529.9 | {21422., 41270.} | 61.0033 | 0.421313 |
| 37 | newly 66 weeks cumul | 88408. | 80614.6 | {53459., 93555.} | 12.534 | 0.285053 |
| 38 | newly 66 weeks cumul | 81546. | 14954.8 | {0., 31176.} | 81.6609 | 2.22106 |
| 39 | newly 60 weeks cumul | 70294. | 88050.1 | {83253., 93287.} | 25.6777 | 0.0986692 |
| 40 | newly 61 weeks cumul | 84270. | 39567.7 | {34040., 43179.} | 53.0466 | 0.329891 |
| 41 | newly 62 weeks cumul | 72211. | 45432.5 | {41135., 47358.} | 37.0837 | 0.202161 |
| 42 | newly 66 weeks cumul | 73182. | 69014.7 | {42026., 92872.} | 25.4562 | 0.33844 |
| 43 | newly 59 weeks cumul | 92457. | 87990.8 | {84566., 89353.} | 4.83054 | 0.0216494 |
| 44 | newly 65 weeks cumul | 91937. | 88281.1 | {83005., 93568.} | 4.63737 | 0.0212683 |
| 45 | newly 64 weeks cumul | 82950. | 84509.4 | {78463., 92448.} | 9.19254 | 0.125848 |
| 46 | newly 64 weeks cumul | 85120. | 88811.5 | {78636., 93217.} | 8.12269 | 0.0360061 |
| 47 | newly 66 weeks cumul | 82631. | 82755.7 | {73710., 92769.} | 11.0822 | 0.233388 |
| 48 | newly 66 weeks cumul | 91693. | 83357.2 | {70793., 93619.} | 9.89452 | 0.198807 |
| 49 | newly 68 weeks cumul | 88820. | 43831.9 | {0., 92518.} | 52.1871 | 2.75274 |
| 50 | newly 59 weeks cumul | 91561. | 88127.5 | {85202., 90557.} | 3.78451 | 0.0168858 |
